# Editorial: Focus on methods: neural algorithms for bio-inspired robotics

**DOI:** 10.3389/fnbot.2023.1250645

**Published:** 2023-07-25

**Authors:** Luca Patanè, Guoping Zhao

**Affiliations:** ^1^Department of Engineering, University of Messina, Messina, Italy; ^2^Lauflabor Locomotion Laboratory, Centre for Cognitive Science, Technical University of Darmstadt, Darmstadt, Germany

**Keywords:** neural control, bio-robotics, biological signal processing, machine learning, intelligent machines

## 1. Introduction

Bio-inspired robotics aims to develop robotic systems that mimic or are inspired by biological systems and processes. One of the main challenges in this field is the development of neural algorithms that enable robots to exhibit intelligent behavior and interact effectively with their environment. Neural algorithms use the principles of neural computation and machine learning to solve complex problems and enable robots to perform tasks with greater efficiency, adaptability, and autonomy. In recent decades, research has gained deep insights into biological systems, from cell biology to physiology, biomechanics, and locomotion (Meyer and Guillot, 2008; Mazzolai et al., 2020; Valero-Cuevas and Erwin, 2022). As biological understanding advances, so does the field of robotics and bio-inspired robotics. The way animals interact with the world through object manipulation, locomotion and spatial navigation can be applied to the embodied intelligence of robots to enable better performance. Knowledge of the neural mechanisms underlying decision-making processes is constantly evolving as shown, for example, by interesting international projects mapping the nervous system of the fruit fly (Court et al., 2023; Naddaf, 2023). A recent review of insect-inspired robots shows the useful influence of biological systems at different levels of robot design, from the mechanical part to the locomotion control system to the sophisticated capabilities involving decision-making processes (Manoonpong et al., 2021). The influence of biological systems in the development of control architectures is enormously increasing and includes hardware-oriented solutions (Chen et al., 2019; Linares-Barranco et al., 2020). Robotics and neuroscience are closely related fields that aim to understand how autonomous agents can achieve agile, efficient, and robust locomotion. Robotics has already gained valuable insights from studying animals and applying neuromechanical principles. Similarly, neuroscience has benefited from the tools and ideas of robotics to explore how the body, physical interactions with the environment and sensory input influence animal behavior. This two-way exchange between the two disciplines was recently reviewed in Ramdya and Ijspeert (2023). This Research Topic aims to present the neural algorithm problem for bio-inspired robotics, focusing on the integration of neural algorithms and computation in robot bodies and the resulting interaction with the environment. Several notable applications in this area were considered and summarized here.

Computational models are important tools for extracting information from complex data such as biological signals. In Mechtenberg and Schneider, an application is presented that focuses on estimating the position of the innervation zone of motor units using a computational sEMG model. The method uses a wavelet-based algorithm to localize the potentials of motor units and accurately estimate the position of the center of the innervation zone.

Machine learning techniques are widely used in the biomedical field to advance the integration of robotic systems into therapeutic protocols. In the context of stroke rehabilitation, a method for redesigning the human-robot interaction space based on patients' recovery states (Li et al.). Machine learning models, including a PSO-SVM classification model and an LSTM-KF regression model, are used to detect motor skills and achieve assistance-as-needed (AAN) control. Due to the massive use of sensors to improve the perceptual capabilities of robots, multimodal information fusion is a key element that needs further research to improve robot recognition. Industrial applications of robots today are oriented toward tasks that require decision-making processes and high precision. Ding et al. addresses the challenge of recognizing multimodal systems through adaptive visual-tactile fusion. The proposed method improves the recognition capability of robots by converting tactile sequences into images and using a visual-tactile fusion network that achieves a classification accuracy of 99.3%. The use of bioinspired solutions to develop smart sensing devices is a growing area of research (Tayarani-Najaran and Schmuker, 2021). Event-based cameras are being used to either dramatically improve frame rates or reduce data transfer in applications that require real-time conditions. Annamalai et al. proposed an architecture for video anomaly detection using sparse submanifold convolutional networks. The proposed architecture, which includes a conditional generative adversarial network and a deep-learning memory layer, achieves good performance with lower computational complexity.

Robotic applications increasingly require real-time sensory signal processing solutions to solve problems such as collision detection and avoidance. To improve collision detection capabilities, a direction-selective visual neural network based on the fractional order differential operator is proposed (Wang et al.). This network not only detects collision hazards but also determines the direction of the object's movement, mimicking the response characteristics of biological neurons. Following the same path, Luan et al. proposes a neural network model inspired by the Monostratified Lobula Giant type1 neurons in the crab's visual system to determine the spatial position of approaching objects. The model successfully encodes and conveys spatial information, demonstrating its potential for developing intelligent machines that can interact with dynamically changing environments.

In summary, neural algorithms play a crucial role in the advancement of bio-inspired robotics by enabling robots to exhibit intelligent behavior and interact effectively with their environment. The applications discussed in this article demonstrate the potential of neural algorithms in various aspects of bio-inspired robotics, including motor unit innervation zone estimation, adaptive fusion detection, rehabilitation training assistance, collision-sensitive visual networks, spatial localisation networks, and event-based anomaly detection. These applications highlight the effectiveness and versatility of neural algorithms in improving the capabilities of robots and developing intelligent machines. Continued research and advances in this field promise further innovations and breakthroughs in bio-inspired robotics.

## Author contributions

All authors listed have made a substantial, direct, and intellectual contribution to the work and approved it for publication.

## References

[B1] Chen G. Bing Z. Röhrbein F. Conradt J. Huang K. Cheng L.. (2019). Toward brain-inspired learning with the neuromorphic snake-like robot and the neurorobotic platform. IEEE Trans. Cogn. Dev. Syst. 11, 1–12. 10.1109/TCDS.2017.2712712

[B2] Court R. Costa M. Pilgrim C. Millburn G. Holmes A. McLachlan A.. (2023). Virtual Fly Brain—An interactive atlas of the Drosophila nervous system. Front. Physiol. 14, 1076533. 10.3389/fphys.2023.1076533 36776967 PMC9908962

[B3] Linares-Barranco A. Perez-Peña F. Jimenez-Fernandez A. Chicca E. (2020). ED-biorob: a neuromorphic robotic arm with fpga-based infrastructure for bio-inspired spiking motor controllers. Front. Neurorobot. 14, 590163. 10.3389/fnbot.2020.590163 33328951 PMC7735321

[B4] Manoonpong P. Patanè L. Xiong X. Brodoline I. Dupeyroux J. Viollet S.. (2021). Insect-inspired robots: Bridging biological and artificial systems. Sensors 21, 7609. 10.3390/s21227609 34833685 PMC8623770

[B5] Mazzolai B. Tramacere F. Fiorello I. Margheri L. (2020). The bio-engineering approach for plant investigations and growing robots. A mini-review. Front. Robot. AI 7, 573014. 10.3389/frobt.2020.573014 33501333 PMC7806088

[B6] Meyer J.-A. Guillot A. (2008). “Biologically Inspired Robots,” in Springer Handbook of Robotics eds. B., Siciliano, O., Khatib (Berlin, Heidelberg: Springer). 10.1007/978-3-540-30301-5_61

[B7] Naddaf M. (2023). Gigantic map of fly brain is a first for a complex animal. Nature 615, 571. 10.1038/d41586-023-00709-7 36899190

[B8] Ramdya P. Ijspeert A. J. (2023). The neuromechanics of animal locomotion: From biology to robotics and back. Sci. Robot. 8, eadg0279. 10.1126/scirobotics.adg0279 37256966

[B9] Tayarani-Najaran M.-H. Schmuker M. (2021). Event-based sensing and signal processing in the visual, auditory, and olfactory domain: A review. Front. Neur. Circ. 15, 610446. 10.3389/fncir.2021.610446 34135736 PMC8203204

[B10] Valero-Cuevas F. Erwin A. (2022). Bio-robots step towards brain body co-adaptation. Nat. Mach. Intell. 4, 737–738. 10.1038/s42256-022-00528-x

